# In Silico Docking and Spectroscopic Evaluation of a Thiocarbohydrazone Derivative: Structural Elucidation and Enzyme Inhibitory Mechanisms

**DOI:** 10.3390/ph19071108

**Published:** 2026-07-17

**Authors:** Maria Karatzia, Nikitas Georgiou, Ektoras Vasileios Apostolou, Eleftherios Papamichalis, Sophia C. Hayes, Thomas Mavromoustakos, Demeter Tzeli

**Affiliations:** 1 Department of Chemistry, University of Cyprus, P.O. Box 20537, Nicosia 1678, Cyprus; karatzia.d.maria@ucy.ac.cy (M.K.);; 2 Center for Interdisciplinary Biosciences, Technology and Innovation Park, P.J. Safarik University in Kosice, Jesenna 5, 04001 Kosice, Slovakia; 3 Laboratory of Organic Chemistry, Department of Chemistry, National and Kapodistrian University of Athens, Panepistimiopolis Zografou, 15771 Athens, Greece; 4 Laboratory of Physical Chemistry, Department of Chemistry, National and Kapodistrian University of Athens, Panepistimioupolis Zografou, 11571 Athens, Greece; 5 Theoretical and Physical Chemistry Institute, National Hellenic Research Foundation, 48 Vassileos Constan-tinou Ave., 11635 Athens, Greece

**Keywords:** thiocarbohydrazone, molecular docking, DFT, IR/raman analysis

## Abstract

**Objectives:** Thiocarbohydrazones represent an important class of Schiff base derivatives with versatile chemical and biological properties. **Methods:** Herein, we present a combined in silico spectroscopic and molecular docking investigation of N′-benzylidenehydrazinecarbothiohydrazide (1). **Results:** Conformational docking studies were conducted against cathepsin B, acetylcholinesterase, HER2, protein kinase C, and protein kinase A. The compound displayed favorable binding affinities and key interactions within the catalytic sites of all targets, with the strongest predicted binding observed for acetylcholinesterase. Notably, all conformers exhibited higher affinity for protein kinase C than the reference inhibitor balanol, and hydroxylation led to an approximately 10% enhancement in docking performance. Density functional theory (DFT) calculations were employed to analyze vibrational properties, and IR and Raman spectra were computed to elucidate structural features and conformational behavior. **Conclusions**: The integrated spectroscopic and docking analyses provide mechanistic insights into ligand–target interactions and support rational drug design. These findings identify thiocarbohydrazone derivatives as promising multi-target candidates for the development of enzyme inhibitors relevant to neurodegenerative, oncological, and inflammatory diseases.

## 1. Introduction

Compounds containing sulfur and nitrogen have attracted considerable attention in recent years due to their diverse pharmacological potential and drug-like properties [1]. Among these, thiocarbohydrazones have emerged as particularly promising, owing to their distinctive structural features and versatile coordination and biological properties.

Thiocarbohydrazones belong to the broader class of Schiff base derivatives, which have long been pivotal in medicinal chemistry and pharmaceutical development. Extensive studies have demonstrated that Schiff bases and their metal complexes exhibit a wide spectrum of biological activities, including antimicrobial, antifungal, antiviral, antioxidant, and anticancer effects [2,3]. First synthesized in 1925, thiocarbohydrazones can be structurally classified as symmetric or asymmetric, providing a flexible scaffold for ligand design. Their ability to coordinate metals via the sulfur atom and the imine nitrogen arises from the tautomeric equilibrium between the thione (thioketo) and thiol (thioenol) forms, a property that underpins their biological versatility.

Historically, thiocarbohydrazones were among the first antiviral compounds identified, exhibiting activity against diverse DNA and RNA viruses. Despite their structural similarity to thiosemicarbazones—which have been extensively explored for anticancer and other bioactivities—thiocarbohydrazones remain comparatively under-investigated [1,4]. This gap is surprising, given their potential as multifunctional pharmacophores.

The biological activity of hydrazone and thiocarbohydrazone derivatives is strongly influenced by their molecular conformation, as different conformers may exhibit distinct affinities toward biological targets despite having similar intrinsic stabilities. Previous quantum chemical studies have demonstrated that conformational analysis can significantly affect the electronic properties and preferred binding modes of hydrazone-based ligands. This has the result of highlighting the importance of conformational analysis in understanding structure–activity relationships and molecular recognition [5].

Thiosemicarbazones (TSCs) and thiocarbohydrazones (TCHs) are classes of sulfur- and nitrogen-containing compounds that have attracted significant scientific attention because of their broad spectrum of biological and physicochemical properties. Their molecular structures contain donor atoms capable of forming stable coordination complexes with transition metals, which contributes to their remarkable pharmacological and biochemical activities. In recent years, these compounds have been described in literature and investigated using both spectroscopic techniques and in silico approaches. Among these methods, NMR spectroscopy and conformational analysis play a crucial role in elucidating the structural behavior of organic molecules, offering valuable information about tautomeric balance, hydrogen-bonding interactions, and molecular flexibility in solution.

Beyond their biological properties, numerous studies have demonstrated that thiosemicarbazones and thiocarbohydrazones can be modified through the introduction of various functional groups, significantly influencing their reactivity and therapeutic potential. Different aromatic, heterocyclic, halogenated, alkyl, and hydroxyl substituents have been associated with enhanced biological performance and improved stability of these compounds. For example, the incorporation of electron-donating or electron-withdrawing groups into the molecular framework has been shown to alter metal-binding affinity, lipophilicity, and cytotoxic activity. Additionally, Schiff base derivatives [6] containing pyridine, quinoline, and benzaldehyde moieties have exhibited increased antimicrobial and anticancer activities, demonstrating the strong relationship between structural modification and bioactivity [7,8].

Furthermore, researchers have associated thiosemicarbazones and thiocarbohydrazones with a wide range of hybrid molecular systems aimed at improving pharmacological efficiency and selectivity [9,10]. The combination of these compounds with heterocyclic rings, ferrocene units, carbohydrate fragments, and metal complexes [11] has resulted in derivatives with enhanced therapeutic properties and reduced toxicity. Consequently, ongoing investigations into the structural diversity of thiosemicarbazones and thiocarbohydrazones continue to provide valuable insights into their potential applications in medicinal chemistry, coordination chemistry, and material science.

Building upon our previous studies on thiosemicarbazone and thiocarbohydrazone [12,13], this study combines computational and theoretical approaches to evaluate a novel thiocarbohydrazone derivative. Using Density Functional Theory (DFT) [14,15,16], we calculate IR and Raman spectra, employing both “harmonic” [17] and “anharmonic” [18] treatments to capture the vibrational characteristics critical for spectroscopic interpretation [19,20]. To assess biological potential, molecular docking was performed against targets with established anticancer and antineurological relevance [1,21], providing insight into possible binding modes and affinities. Moreover, a hydroxyl-functionalized thiocarbohydrazone derivative was designed to probe whether targeted modification could enhance predicted binding and biological activity. Complementary Absorption, Distribution, Metabolism, Excretion (ADME) analyses indicate favorable drug-likeness and safety profiles, highlighting the compound’s potential for future experimental validation.

## 2. Results

### 2.1. DFT Calculations

**Figure 1:**
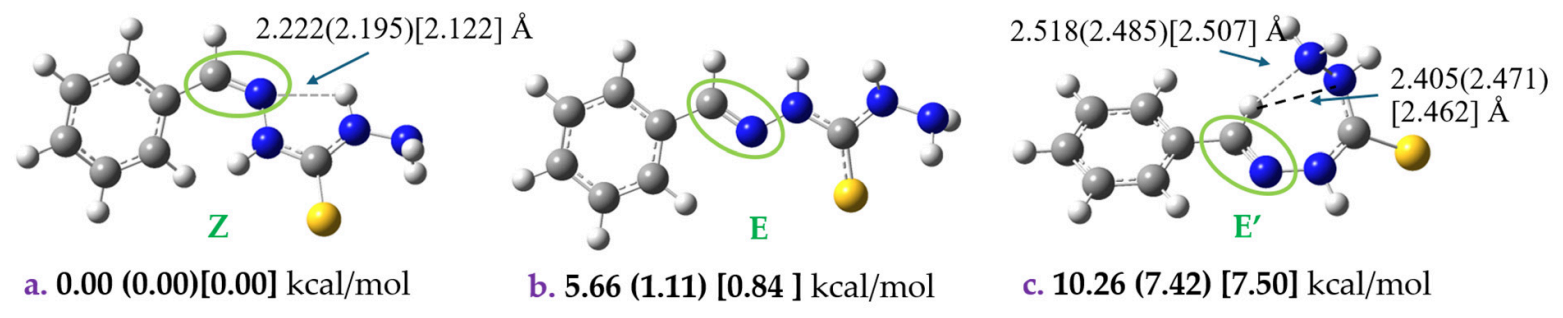
Calculated conformers of compound *N*-benzylidenehydrazinecarbo-thiohydrazide (1) at (**a**) B3LYP/6-311+G(d,p). Relative energies at the gas phase (**b**) in DMSO solvent) [(**c**) in water solvent] are provided.

**Geometry:** The geometry of the lowest energy structures of the *N*-benzylidenehydrazinecarbo-thiohydrazide (1) molecule and the corresponding IR and Raman spectra were studied via the B3LYP/6-311+G (d,p) methodology in the gas phase, in DMSO solvent, and in water solvent. At first, a preliminary conformational analysis using the AutoMeKin code was carried out to find the lowest energy structures [22]. Then the lowest energy conformers were calculated at the DFT level of theory, see Figure 1.

The most energetically stable conformation is conformer **a**, stabilized by a strong intramolecular N–H···N hydrogen bond (2.122 Å in water solvent), which reinforces molecular rigidity. Conformer **c** is also stabilized through two hydrogen-bonding interactions (2.462 Å and 2.507 Å in water solvent) involving the terminal amino groups and the bridging C–H hydrogen, whereas conformer **b** lacks any intramolecular hydrogen bonding, resulting in lower stability. The **a** and **b** structures correspond to the Z and E conformers with respect to the C=N geometry, and they differ only by 1.11 (0.84) kcal/mol in DMSO (water) solvent. The **c** structure is also an E conformer, but the –CH-thiohydrazide forms a seven-membered ring, see Figure 1.

**IR and Raman spectra**: Computational analysis of infrared (IR) and Raman spectra provides critical insights into molecular vibrations and serves as a robust validation of structural features. IR spectroscopy probes vibrational modes through light absorption, facilitating the identification of functional groups (e.g., –OH, –C = O) and bond types (e.g., C–H, N–H), whereas Raman spectroscopy, based on monochromatic light scattering, offers complementary vibrational information. Incorporating anharmonic IR calculations significantly enhances frequency predictions by accounting for deviations from ideal harmonic behavior, yielding a more realistic representation of molecular dynamics and enabling high-precision comparison with experimental data.

**Scheme 1:**
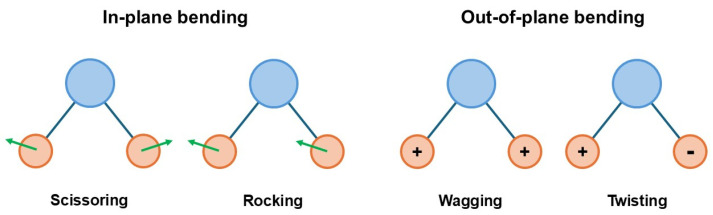
Vibrational modes; τ = Twisting, γ = Wagging, ρ = Rocking, and δ = Scissoring.

**Figure 2:**
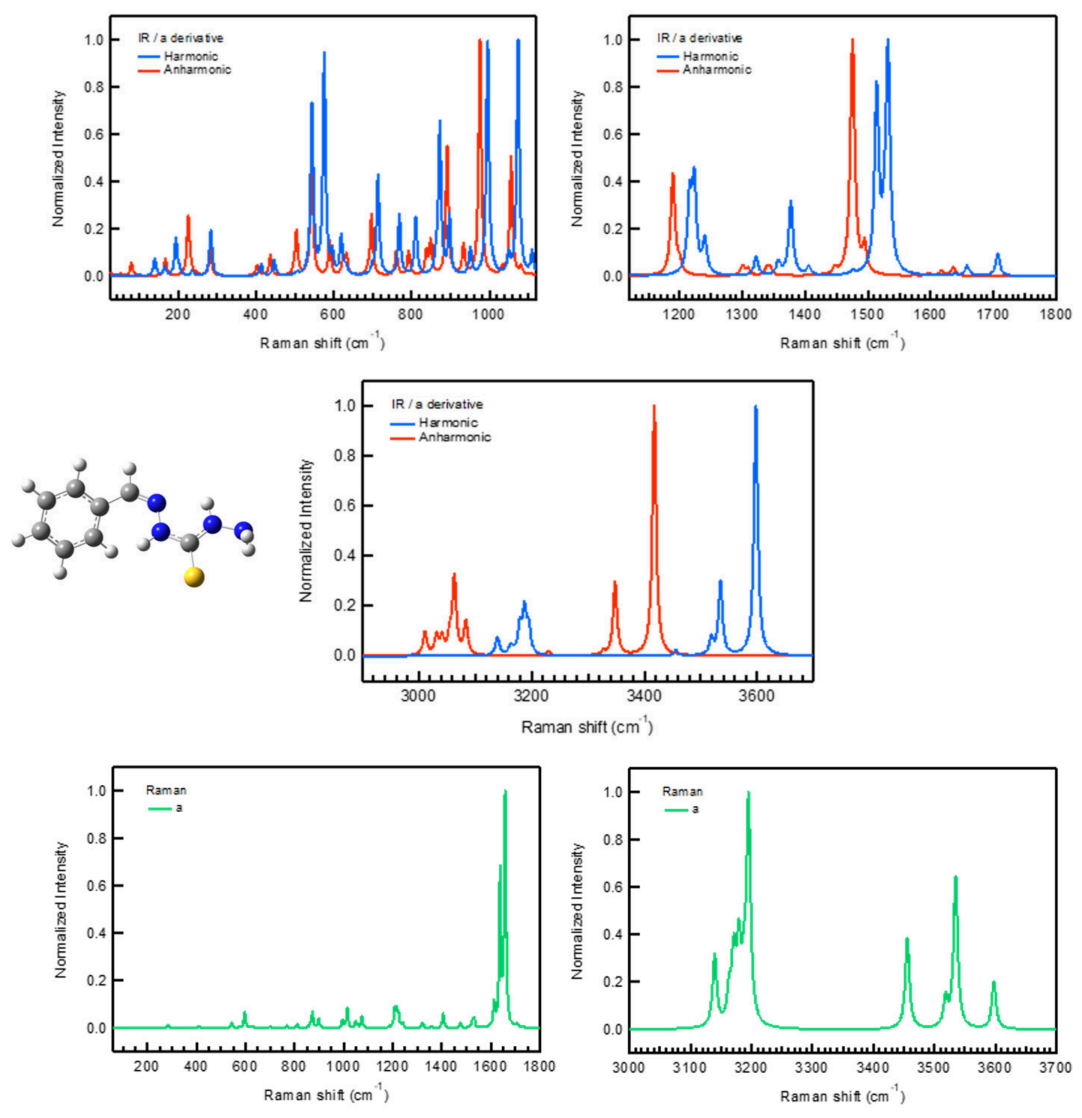
Theoretical IR (harmonic and anharmonic) and harmonic Raman spectra of conformer **a**.

**Figure 3:**
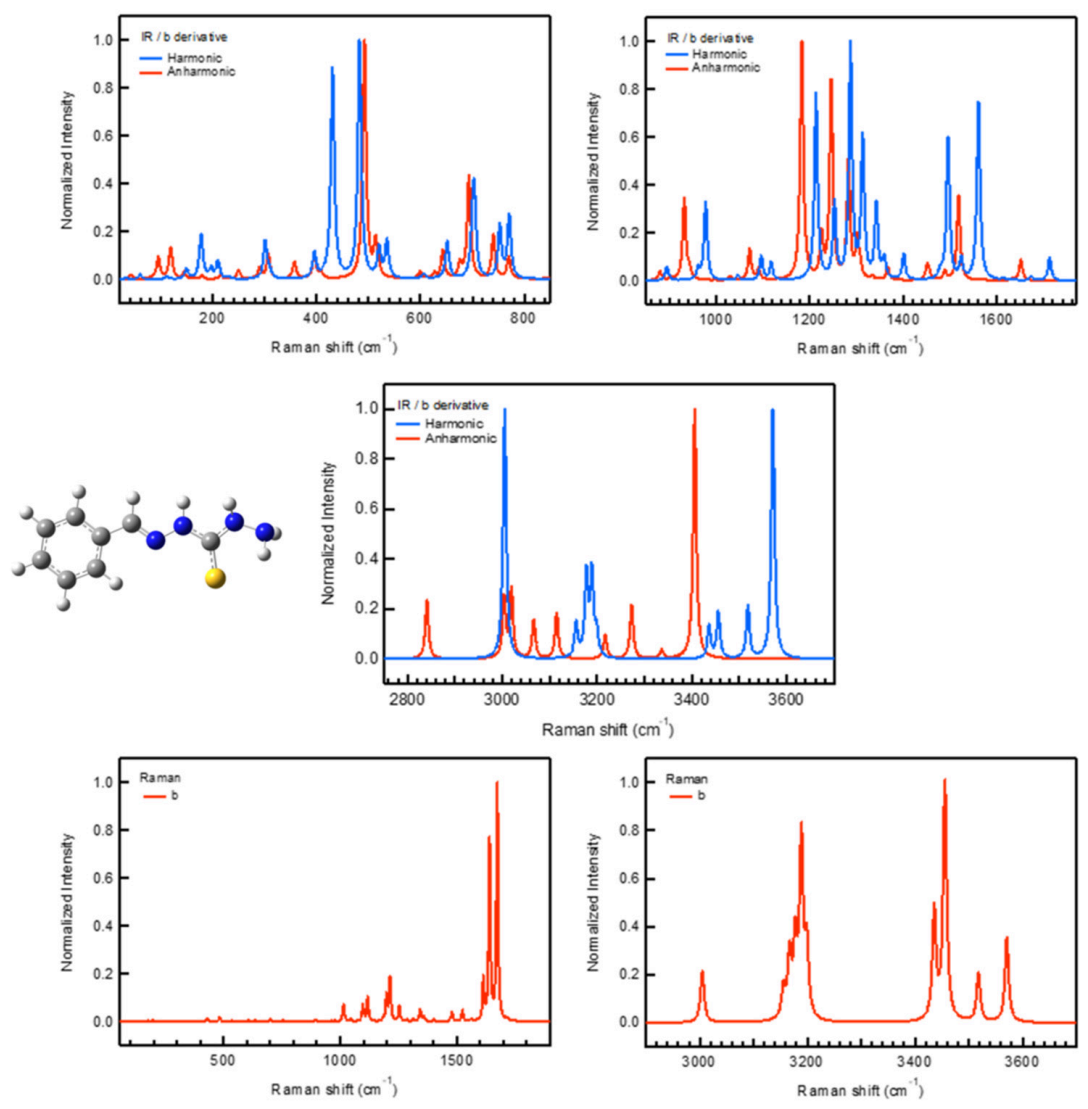
Theoretical IR (harmonic and anharmonic) and harmonic Raman spectra of conformer **b**.

**Figure 4:**
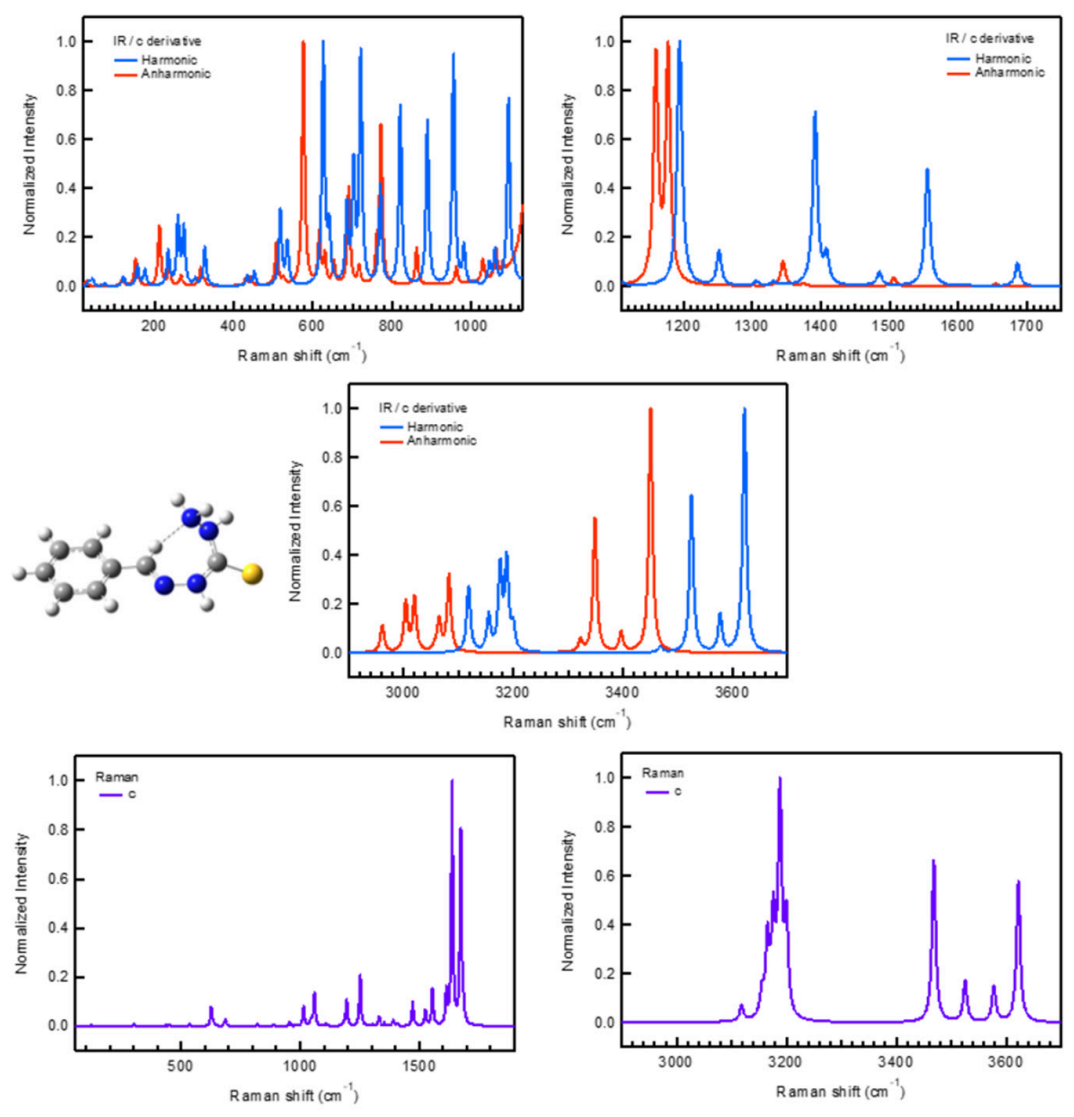
Theoretical IR (harmonic and anharmonic) and harmonic Raman spectra of conformer **c**.

The harmonic and anharmonic IR spectra, along with the Raman spectra of the a, b, and c conformers, are presented in Figure 2, Figure 3 and Figure 4, while selected harmonic and anharmonic vibrational modes and assignments (Scheme 1) are summarized in Table 1, Table 2 and Table 3. The anharmonic effects were calculated for each vibrational mode via the second-order vibrational perturbation theory (VPT2). VPT2 is a computational method that accounts for anharmonic effects in molecular vibrations, improving accuracy over the harmonic approximation while remaining computationally efficient.

In general, the inclusion of anharmonicity results in red shifts of the harmonic vibrational modes up to 192 cm^−1^ for the a conformer and 219 cm^−1^ for the b one. For specific modes, the anharmonicity results in blue shifts in the case of the a and b conformers up to −53 cm^−1^. This mode corresponds to ρ (NH2) motion, where the harmonic vibrational mode of 194 (196) cm^−1^ shifts to 246 (249) cm^−1^ for the a (b) conformers. On the contrary, for the c conformer, the anharmonicity results only in red shifts.

**Figure 5:**
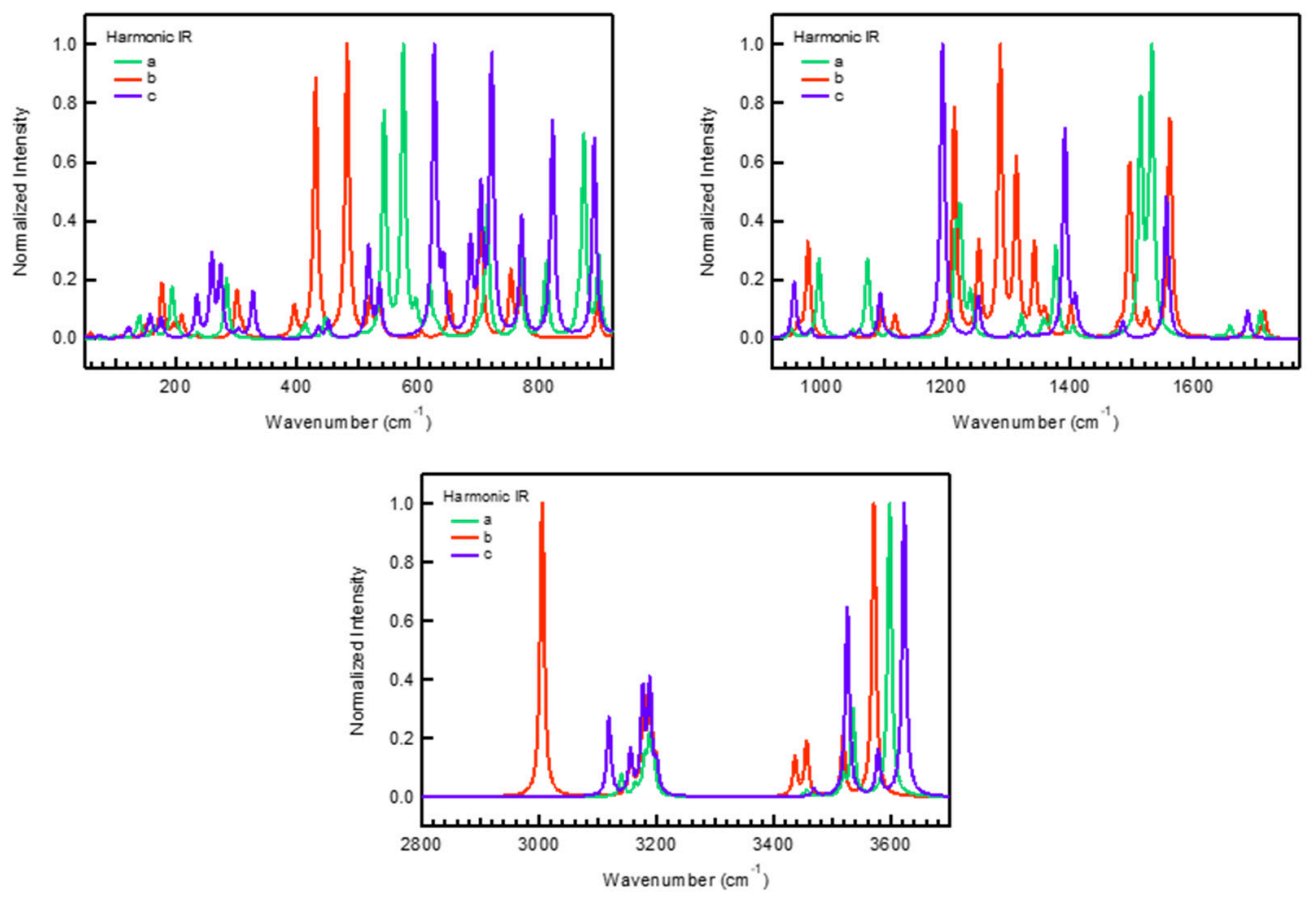
Superposition of normalized harmonic IR spectra for the three molecular configurations (**a**–**c**).

**Comparison:** The calculated harmonic IR spectra of conformers a, b, and c (Figure 5) exhibit both common vibrational features and subtle conformer-dependent differences that reflect variations in intramolecular interactions and structural stability. All three conformers display characteristic bands associated with the thiocarbohydrazone scaffold, including aromatic C–H stretching modes in the 3000–3100 cm^−1^ region and strong bands in the 1500–1650 cm^−1^ region corresponding to C=N and aromatic C=C stretching vibrations. Nevertheless, noticeable differences in band positions and intensities are observed, particularly for modes involving the hydrazone N–H and thiocarbonyl groups, which are highly sensitive to intramolecular hydrogen bonding and conformational rearrangements, i.e., differences of about 50 cm^−1^ are observed at the 3500–3600 cm^−1^ region. For instance, the N–H stretching of =N–N–H group is observed at 3571 cm^−1^ for b and 3622 cm^−1^ for c. Furthermore, the C–H stretching band that corresponds to the hydrogen involved in a thiocarbohydrazone ring in **c** is found at 3119 cm^−1^, while in b this is at 3004 cm^−1^. Thus, the highest energy of the vibrational modes at **c** is due to the fact that the ring is formed and the vibration is constricted. Overall, these spectral variations highlight the role of intramolecular hydrogen bonding in stabilizing specific conformations, which may also influence their preferred bioactive conformations in molecular docking studies.

Furthermore, low-frequency skeletal vibrations (<1000 cm^−1^) show distinct patterns among the three conformers, arising from different torsional arrangements of the aromatic and hydrazone fragments. These variations further confirm that conformational changes significantly influence vibrational coupling and molecular rigidity. For instance, an intense double peak of b at 420 and 480 cm^−1^ is shifted to 520 and 540 cm^−1^ respectively at **c.** Note also that the wagging of the phenyl ring is observed at 446 cm^_1^ for **a** and 520 cm^−1^ for b and c.

The effects of anharmonicity are particularly pronounced in high-frequency vibrational modes, especially those involving light atoms (e.g., C–H, N–H, and O–H stretches). Accounting for this enhanced anharmonicity is essential for accurate modeling of high-frequency vibrations, as it directly affects both spectral line positions and intensities. The observed differences between harmonic and anharmonic IR spectra can be correlated with the binding affinities of the conformers. A strong anharmonic peak in one conformer relative to another reflects greater deviations from the harmonic approximation in its vibrational potential energy surface, often associated with increased conformational flexibility or strain. In the context of molecular docking, rigid or low-strain conformers, which behave more harmonically, typically bind in a more favorable, stable way into enzyme pockets, resulting in more negative docking scores when they fit in that pocket. Conversely, highly flexible conformers with pronounced anharmonicity may incur energy penalties for induced fit when their structure does not fit very well and needs to be adapted.

In the present study, conformers a and b, being more rigid, are expected to fit well into enzyme pockets. Conformer **c**, despite exhibiting strong anharmonic peaks in a narrow spectral region, demonstrates localized flexibility rather than global strain. This selective flexibility may actually facilitate adaptation to diverse enzyme pockets, enhancing binding versatility rather than reducing affinity.

To evaluate the electronic properties of the complexes, the HOMO–LUMO energy difference, ΔE_L-H_, and the reactivity descriptors such as chemical hardness, η = (I − A)/2, softness, σ = 1/η, chemical potential, μ = −(I + A)/2, and electrophilicity, ω = μ^2^/2η were calculated. Note that the energy of the HOMO (E_H_) is directly related to the ionization potential, while the LUMO energy (E_L_) is related to the electron affinity. Therefore, it is considered that I = −E_H_ and A = −E_L_. The calculated frontier molecular orbital (FMO) parameters show only minor differences among the three conformers in the gas phase, DMSO, and water (Table 4 and Table S4 of Supplementary Materials). Conformer **c** exhibits the smallest HOMO–LUMO while the **a** shows the largest gap, suggesting greater kinetic stability. Chemical hardness follows the same trend, while softness varies inversely. The electrophilicity values are similar for all conformers, indicating comparable electron-accepting abilities. Overall, both DMSO and water slightly stabilize the electronic structure, but the conformers retain very similar electronic properties across all solvents [23].

Finally, the NBO charges were calculated for the isomers, see Figure S1 of Supplementary Materials. Similar atomic NBO charges are obtained for all conformers. Only small differences up to 0.03 e^−^ are observed. For instance, the H atoms, when forming a hydrogen bond with the N atoms, present an increase up to +0.03 e−. In all cases, the S atom is negativelly charged with a −0.35 e^−^, while the N atoms have charges that range from −0.30 e^−^ to −0.65 e^−^. The largest ones are observed for the terminal N atoms. Overall, the charge distributions assist in the formation of interactions with the biological targets (see below).

### 2.2. Molecular Docking

Molecular docking studies were performed for conformers a, b, and c of compound 1, against four biological targets: Cathepsin B, Acetylcholinesterase, HER2, Protein Kinase C and Protein Kinase. All three conformers exhibited favorable binding energies and interactions with key amino acid residues in the enzyme active sites.

**Figure 6:**
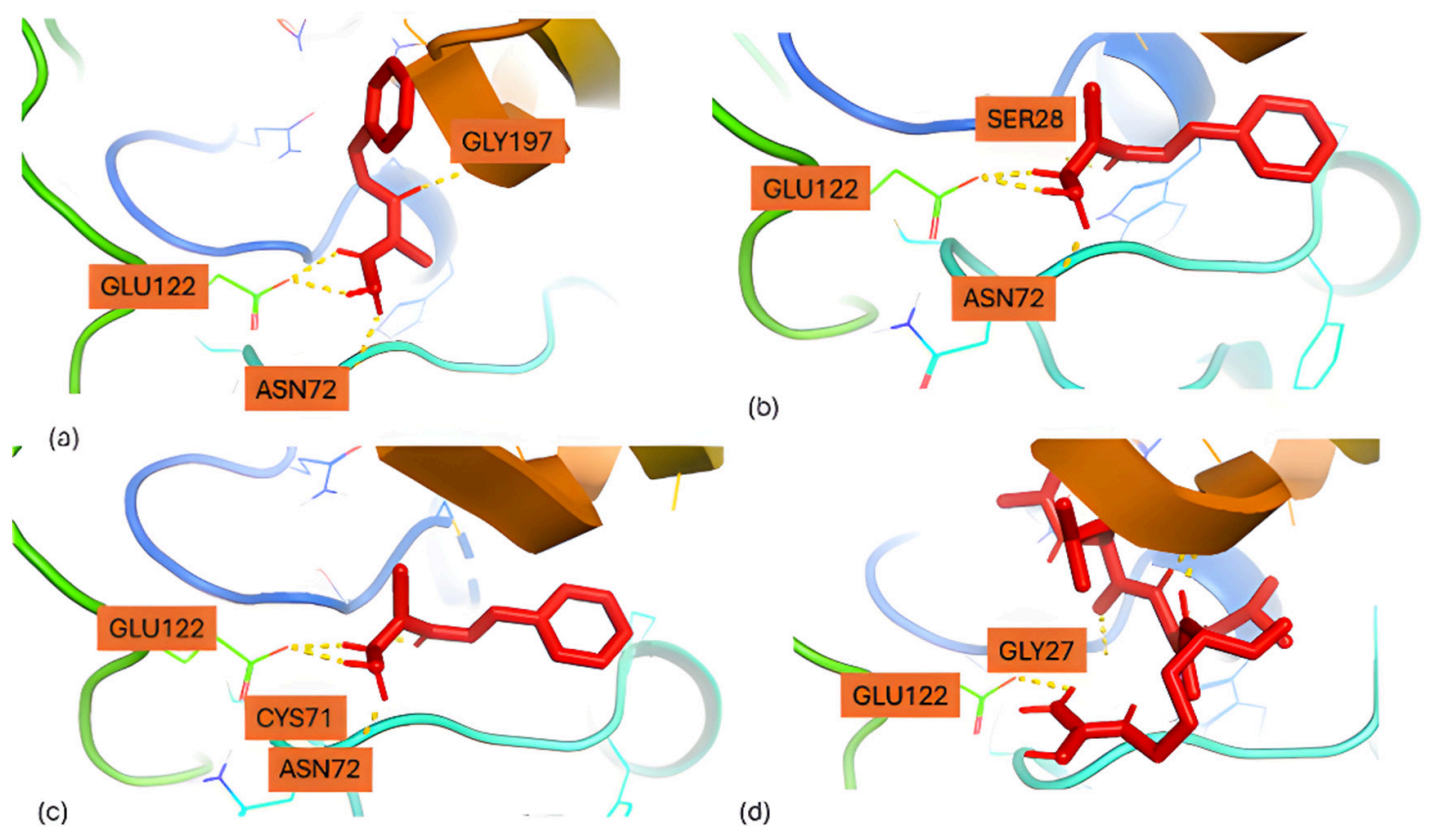
Docking poses of conformers (**a**) **a**, (**b**) **b**, (**c**) **c** and (**d**) **Leupeptin** in the active center of Cathepsin B.

We began our molecular docking analysis by examining interactions of the thiocarbohydrazone derivative with Cathepsin B. Among the three conformers, conformer **c** (Figure 6c) displayed the most favorable (most negative) docking score (−5.21 kcal/mol), reflecting the highest predicted binding affinity, primarily through hydrogen-bonding interactions with GLU122 and ASN72, along with additional stabilization from CYS71. Conformer **a** (Figure 6a) exhibited a similar binding profile, with a docking score of −5.00 kcal/mol, interacting mainly with GLU122 and ASN72, and further stabilized by proximity to GLY197. The overall binding mode closely resembles that of conformer **c**, which explains the similar binding energies. In contrast, conformer **b** (Figure 6b) showed a slightly lower docking score (−4.72 kcal/mol), suggesting reduced binding affinity. Although key interactions with GLU122 and ASN72 were maintained and additional contacts with SER28 were observed, the ligand adopted a less favorable orientation within the binding pocket. These results indicate that while all conformers are capable of engaging the active site, conformers **a** and **c** are better positioned to maximize binding interactions, whereas conformer **b**’s suboptimal pose slightly diminishes its predicted affinity. For comparison, the known Cathepsin B inhibitor, **Leupeptin** (Figure 6d) [24], exhibited a docking score of −5.66 kcal/mol, occupying a similar active-site region and forming hydrogen bonds with GLU122, GLY27, and HIS199, highlighting that the binding mode of compound **1**, particularly conformer **c**, is comparable to a well-established inhibitor.

**Figure 7:**
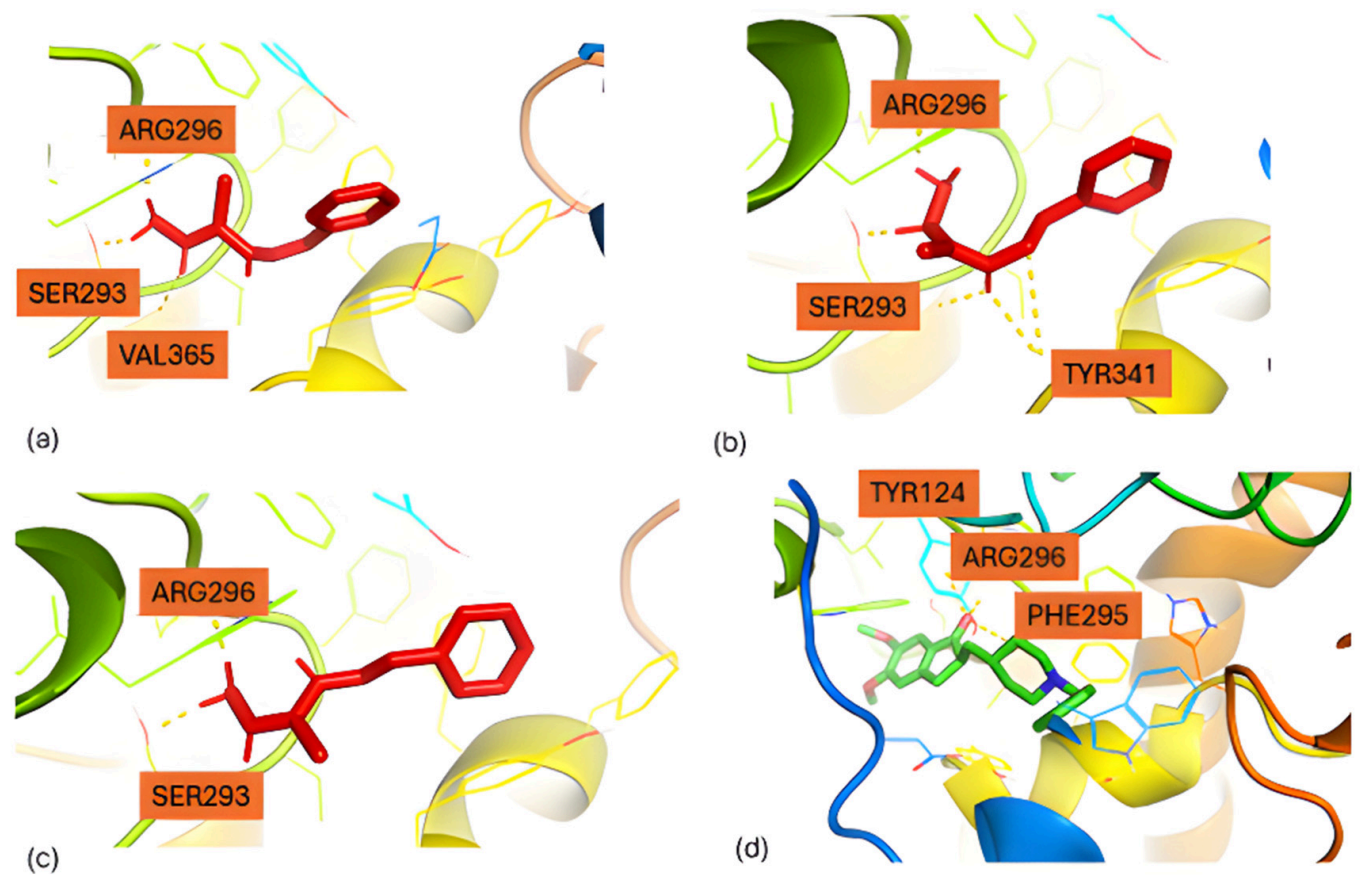
Docking poses of conformers (**a**) **a**, (**b**) **b**, (**c**) **c** and (**d**) **Donepezil** in the active center of Acetylcholinesterase.

Building upon these observations, we next evaluated the binding of the thiocarbohydrazone derivative to Acetylcholinesterase, revealing three highly similar binding conformations with comparable docking scores (Figure 7). The predicted scores were −6.77 kcal/mol (conformer **a**), −6.70 kcal/mol (b), and −6.84 kcal/mol (c), indicating uniformly favorable affinities. In all conformations, the ligand adopts a consistent orientation stabilized primarily through hydrogen bonding interactions with SER293 and ARG296. Additional interactions differentiate the poses: conformer **a** engages in hydrophobic contacts with VAL365, conformer **b** positions its aromatic moiety toward TYR341, forming π–π interactions, and conformer **c**, which achieved the most favorable score (−6.84 kcal/mol), preserves the core hydrogen-bonding network, suggesting a conserved binding mode across conformers. For reference, the well-known Acetylcholinesterase inhibitor **Donepezil** [25] (Figure 7d), exhibited a strong docking score of −11.09 kcal/mol, forming hydrogen bonds with PHE295, ARG296, and TYR124, confirming the reliability of the docking protocol and contextualizing the binding potential of the thiocarbohydrazone derivative.

**Figure 8:**
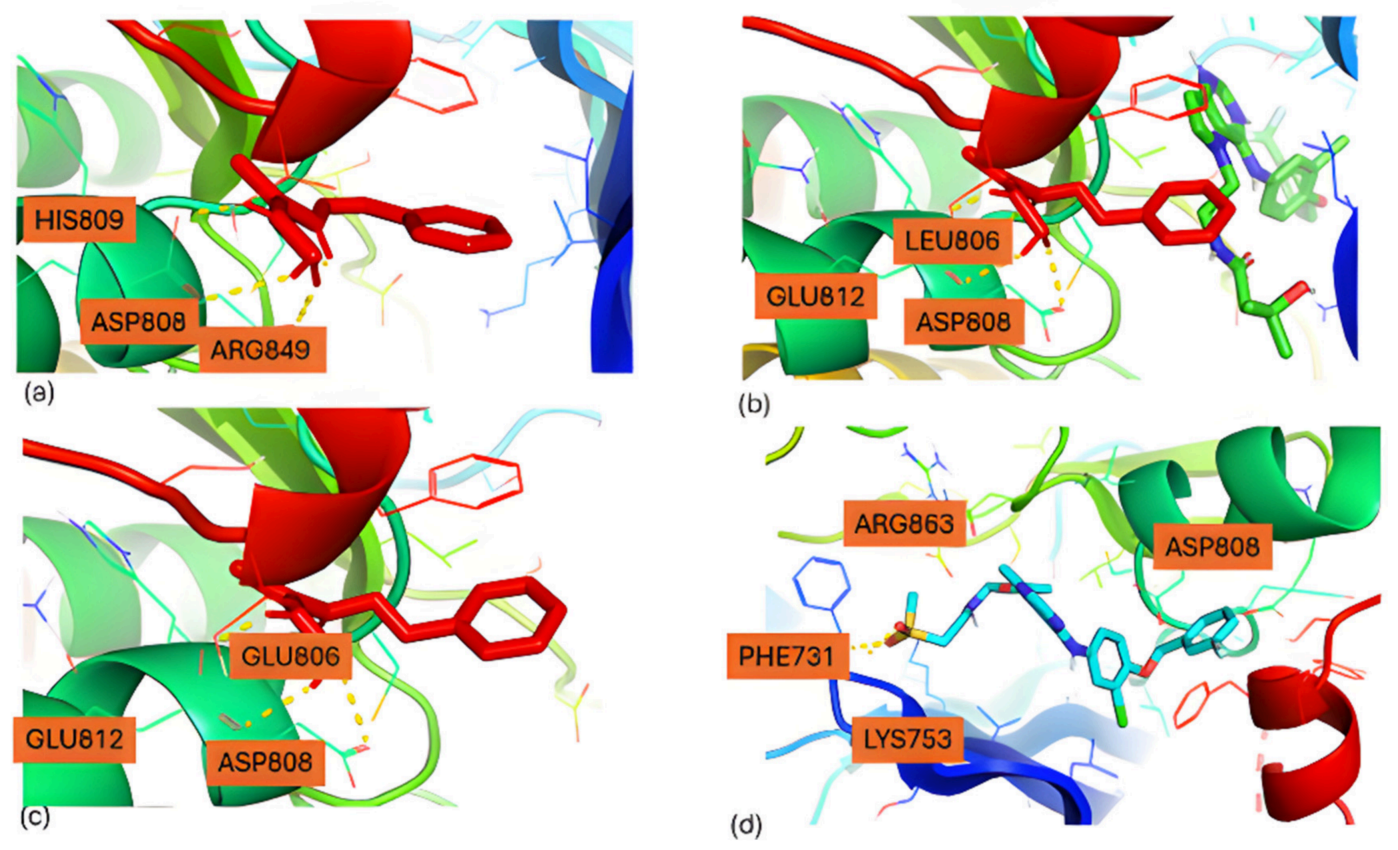
Docking poses of conformers (**a**) **a**, (**b**) **b**, (**c**) **c** and (**d**) **Lapatinib** in the active center of Human Epidermal Growth Factor Receptor 2 (HER2).

To further explore the binding potential of the thiocarbohydrazone derivative across diverse targets, we next investigated interactions with Human Epidermal Growth Factor Receptor 2 (HER2). Docking studies revealed distinct binding affinities among the three conformers (Figure 8). Conformer **b** (Figure 8b) exhibited the most favorable docking score (−7.41 kcal/mol), forming hydrogen-bond and electrostatic interactions primarily with ASP808, GLU812, and LEU806, which likely enhance binding stability. Conformer **c** (Figure 8c), while less favorable (−6.81 kcal/mol), maintains key contacts with ASP808, GLU812, and GLU806, preserving a binding orientation closely resembling that of **b** and suggesting a comparably stable pose within the pocket. In contrast, conformer **a** displayed the lowest score (−6.61 kcal/mol), forming hydrogen bonds mainly with HIS809, ASP808, and ARG849; although it interacts with similar residues, subtle shifts in orientation appear to reduce its predicted affinity relative to b and c. For comparison, the established HER2 inhibitor **Lapatinib** [26] (Figure 8d) demonstrated a docking score of −8.78 kcal/mol, occupying the same active-site region and forming hydrogen bonds with PHE731 and LYS753, providing a benchmark for contextualizing the thiocarbohydrazone derivative’s binding potential.

**Figure 9:**
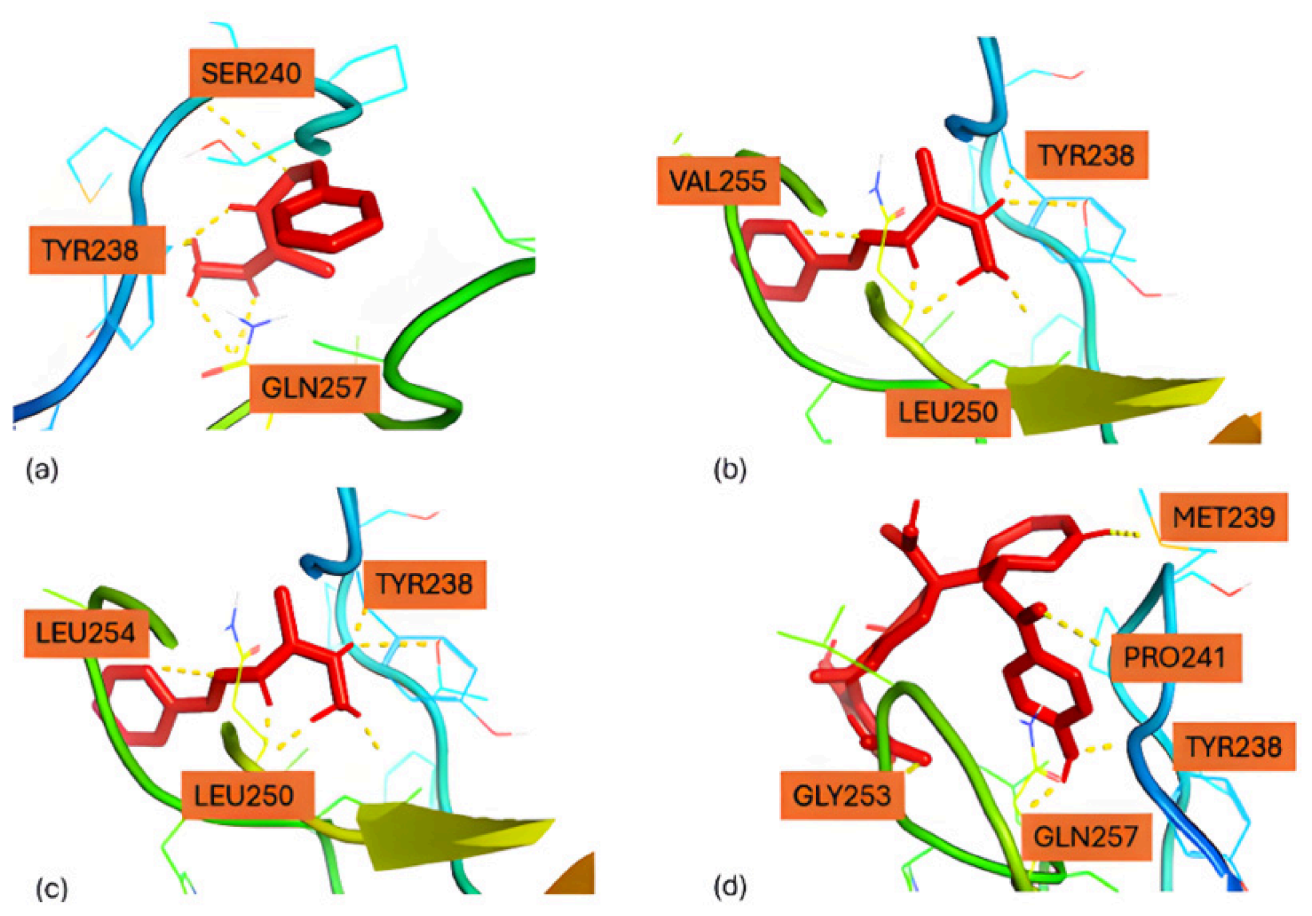
Docking poses of conformers (**a**) **a**, (**b**) **b**, (**c**) **c** and (**d**) **Balanol** in the active center of Protein Kinase C Delta.

Extending our investigation to Protein Kinase C delta (PKC-δ), docking studies revealed that the thiocarbohydrazone derivative adopts favorable binding poses across all three conformers (Figure 9). Conformer **a** (Figure 9a) exhibited a docking score of −5.79 kcal/mol, forming hydrogen-bonding and hydrophobic interactions primarily with TYR238, SER240, and GLN257, indicating that the ligand is well accommodated within the binding pocket. Conformer **b** (Figure 9b) displayed the most favorable score (−5.88 kcal/mol), stabilized through strong interactions with TYR238, VAL255, and LEU250, reflecting enhanced hydrophobic packing and hydrogen bonding. Conformer **c** (Figure 9c) achieved a comparable score (−5.85 kcal/mol), interacting mainly with TYR238, LEU250, and LEU254, suggesting that hydrophobic contacts dominate its binding stabilization. These results indicate that while all three conformers occupy the same active site, conformers b and c are slightly better positioned to maximize stabilizing interactions, whereas conformer **a**, though slightly weaker, still maintains a favorable pose. For reference, the known PKC-δ inhibitor **Balanol** (Figure 9d) [27] exhibited a docking score of −5.62 kcal/mol, engaging key residues TYR238, MET239, PRO241, GLY253, and GLN257, consistent with its established binding mode. Collectively, these results suggest that the thiocarbohydrazone derivative, particularly conformers b and c, can effectively occupy the PKC-δ binding pocket and may serve as promising inhibitors.

**Figure 10:**
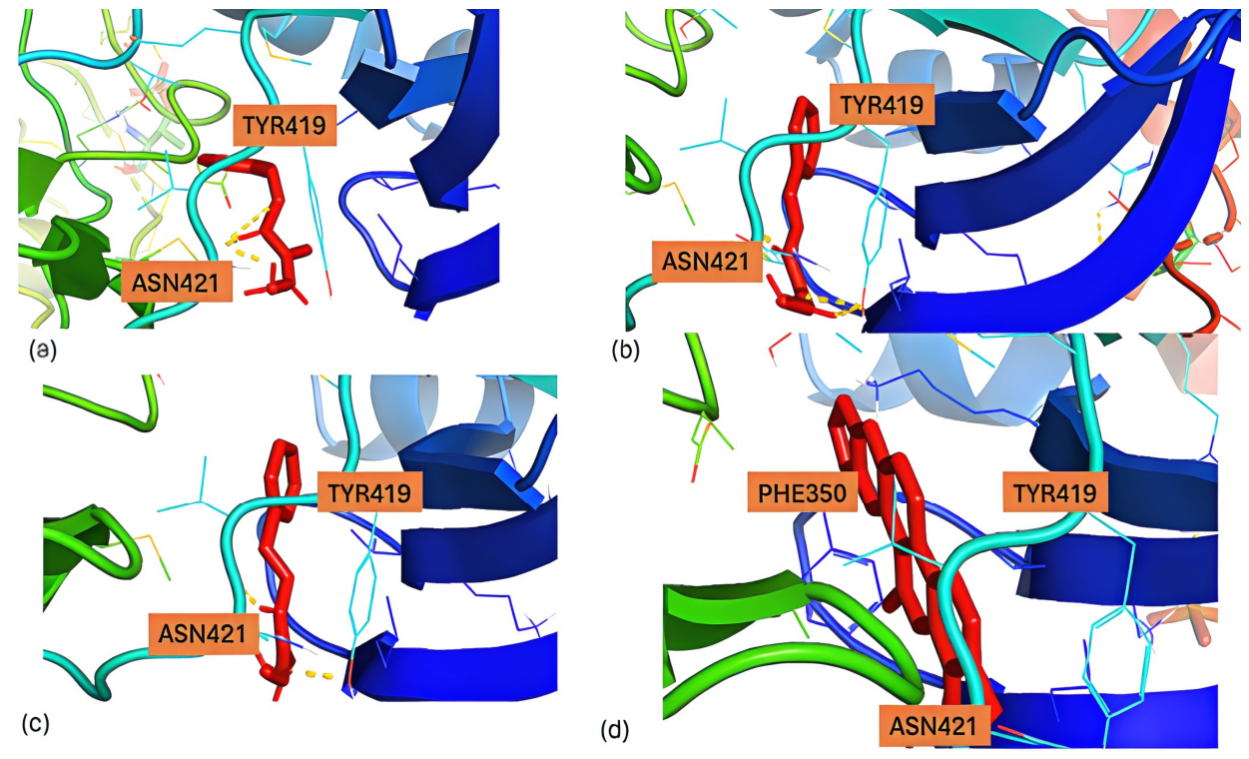
Docking poses of conformers (**a**) **a**, (**b**) **b**, (**c**) **c** and (**d**) **Chelerythrin** in the active center of Protein Kinase C Alpha.

Finally, we extended our docking studies to Protein Kinase C alpha (PKCα) to evaluate the binding potential of the thiocarbohydrazone derivative across additional kinase targets. Conformers a, b, and c exhibited moderate binding affinities, with docking scores of −4.93, −5.40, and −5.04 kcal/mol, respectively (Figure 10a–c). In all three poses, the ligand interacts primarily with TYR419 and ASN421, forming hydrogen-bond and polar interactions that stabilize binding within the catalytic pocket. Although these scores are lower than those observed for the previously investigated enzymes, conformer **b** shows the highest predicted affinity among the three conformers. In contrast, the reference PKCα inhibitor **Chelerythrine** (Figure 10d) [28] displayed a significantly stronger binding score of −9.39 kcal/mol, forming hydrogen-bonding interactions with TYR419, ASN421, and PHE350, consistent with a highly favorable and stable interaction within the catalytic site.

Interestingly, conformer **b**, while not representing the global minimum according to DFT calculations, appears preferentially stabilized in most of the studied enzymes due to favorable intramolecular and intermolecular interactions. Specifically, this conformation is reinforced in each docking pose by an intramolecular N–H···N hydrogen bond, with distances of 2.61 Å in Cathepsin B, 2.47 Å in Acetylcholinesterase, and 2.22 Å in HER2. Moreover, the docking-derived conformations are highly similar to that previously reported for the LOX1 enzyme [13], with the ligand adopting a conserved binding geometry across all investigated targets, suggesting that this pose likely represents the energetically favorable bioactive conformation [13].

**Figure 11:**
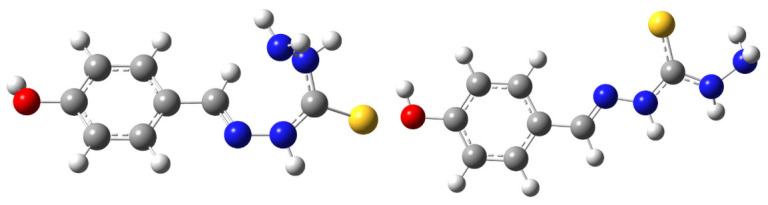
Structure of the **2c** (**left**) and **2b** (**right**) conformation.

Table 5 summarizes the overall molecular docking results, including a comparison with the known reference inhibitors. Additionally, a hydroxyl group was introduced into conformers b and c of the thiocarbohydrazone scaffold (yielding derivatives 2b and 2c) to assess the impact of this modification on binding affinity in the enzymes that gave the least favorable docking scores. The molecular structures of these newly designed derivatives are presented in Figure 11, and the corresponding docking scores are given in Table 5.

**Figure 12:**
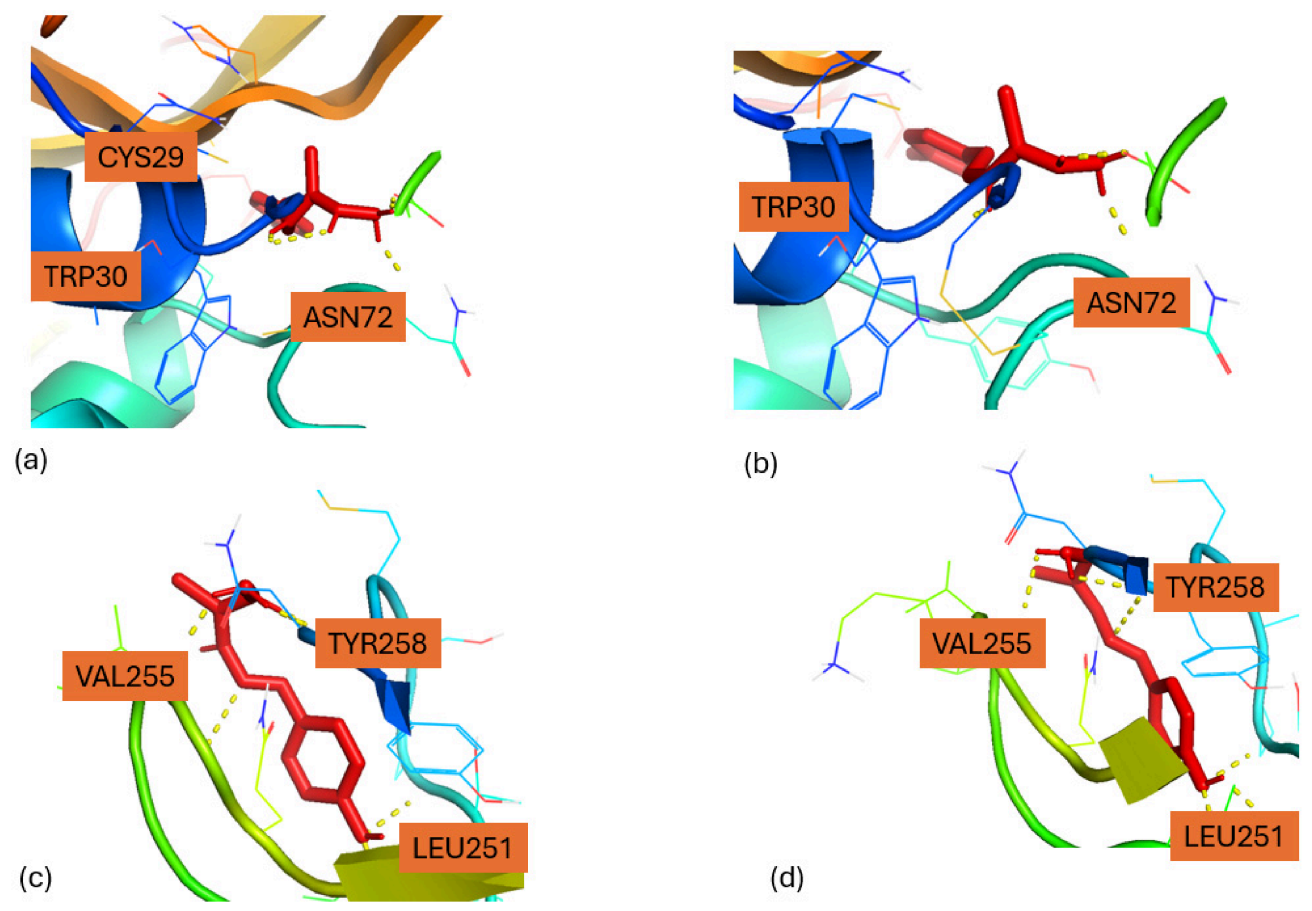
Docking poses of the **2b** (**a**) and **2c** (**b**) conformers in Cathepsin B and **2b** (**c**) and **2c** (**d**) conformers in the active center of Protein Kinase C Delta.

In Cathepsin B, compound 2b (Figure 12a) adopted a stable binding conformation within the active site, forming hydrogen-bond interactions with key residues CYS29 and ASN72, while maintaining π–π stacking interactions with TRP30. Similarly, 2c (Figure 12b) exhibited a comparable binding orientation, forming hydrogen bonds with ASN72 and significant aromatic interactions with TRP30, suggesting a conserved binding mode for the hydroxyl-substituted derivatives.

For Protein Kinase C delta, compound 2b (Figure 12c) occupied the binding pocket and formed multiple stabilizing interactions with hydrophobic residues LEU251 and VAL255, as well as the aromatic residue TYR258. Compound 2c (Figure 12d) displayed a similar binding orientation, engaging in both hydrogen-bonding and hydrophobic interactions with LEU251, VAL255, and TYR258. Overall, the docking poses indicate that hydroxyl substitution does not disrupt the core binding geometry; instead, both derivatives are stabilized within the active site through a synergistic combination of hydrogen bonding and hydrophobic interactions, suggesting that hydroxyl functionalization may enhance ligand–protein interactions and binding adaptability. As a result, compound 2c also binds stronger than the other compounds and also from the known inhibitor balanol.

According to Figure 6, Figure 7, Figure 8, Figure 9, Figure 10, Figure 11 and Figure 12, multiple hydrogen-bonding interactions are formed between the ligands and the enzyme active sites. The predominant interactions correspond to N–H···O and O–H···O hydrogen bonds, which play a key role in stabilizing the ligands within the binding pockets and anchoring them in favorable binding orientations. In addition, weaker C–H···O hydrogen bonds are also observed, in which ligand C–H groups act as hydrogen donors toward protein oxygen atoms. Although individually weaker, these interactions cumulatively contribute to the stabilization of the ligand-enzyme complexes and help define the overall binding geometry. Overall, the docking results suggest that this molecule displays favorable binding affinities toward several biological targets, in agreement with previous reports demonstrating the biological potential of thiocarbohydrazone derivatives against related targets [13,29].

## 3. Materials and Methods

### 3.1. IR and Raman DFT Calculations

Geometry optimizations were performed for three conformers (Figure 1) of (*E*)-*N’*-benzylidenehydrazinecarbothiohydrazide (1), and their theoretical IR and Raman spectra (Figure 2, Figure 3 and Figure 4) were computed using the B3LYP [30,31,32] functional and the 6-311+G(d,p) basis set [33]. The anharmonic effects were calculated for each vibrational mode via the 2nd-order vibrational perturbation theory (VPT2). All quantum chemical calculations were conducted in the gas phase using the Gaussian 16 program [34].

### 3.2. Molecular Docking

Molecular docking experiments were conducted using Autodock software 1.5.6. [35,36]. The DFT conformations of the compounds were taken as the initial structures [13]. After that, molecular docking was carried out using the Lamarckian Genetic Algorithm (LGA) [37]. The protein crystal structures were downloaded from the Protein Data Bank (PDB) and imported directly into AutoDock for analysis. Specifically, the protein structures PDB were 1GMY [38]. 4EY7 [39], 3RCD [40], 8UAK [41], and 1PTR [42].

The target protein structure was prepared using AutoDock Tools. Initially, the protein structure was imported and all crystallographic water molecules, co-crystallized ligands, and non-essential heteroatoms were removed. Missing hydrogen atoms, including polar hydrogens, were added, and Kollman charges were assigned to the protein. The prepared structure was then checked for atom types and saved in PDBQT format, which was used as the receptor input file for the docking calculations. The docking grid was generated around the active/binding site to include the key residues involved in ligand interaction.

Finally, the coordinates from the co-crystallized ligand, which were used for the active center of each macromolecule, were: 1GMY: X = 31.265; Y = 35.384; Z = 35.826, 4EY7: X −18.53; Y = −41.928; Z = 24.258; 3RCD: X = 15.789; Y = 3.2; Z = 24.447; 1PTR: X = 9.456; Y = 24.273; Z = 24.872 and 8UAK: X = 32.38; Y = −8.823; Z = 11.253.

## 4. Conclusions

This study focuses on the in silico investigation of a thiocarbohydrazone derivative (1) using IR and Raman spectroscopy combined with molecular docking simulations. IR and Raman spectra were calculated for Z(a), E(b) and E’(c) conformers of 1 via DFT methodology. In the E’ conformer, a hydrogen bonded ring is formed in thiocarbohydrazone group. The a conformer has the lowest energy structures.

Subsequently, molecular docking studies were performed to identify potential biological targets. The results indicated that Acetylcholinesterase exhibited the most favorable docking scores among the investigated targets. In addition, for Protein Kinase C, all conformations showed stronger predicted binding affinities than the reference inhibitor Balanol.

Although conformer **a** was identified as the global minimum according to the DFT calculations, conformer **c** exhibited the strongest binding affinities in the docking simulations. This observation suggests that the intrinsic stability of an isolated conformer does not necessarily correlate with its binding affinity, since protein–ligand interactions depend on the complementarity between the ligand and the enzyme binding site. The anharmonicity analysis indicates differences in the vibrational behavior of the conformers, with conformers a and b displaying lower overall anharmonicity, consistent with a more rigid structure, whereas conformer **c** exhibits more localized anharmonic features that may reflect greater conformational flexibility. Although these differences may influence the ability of the conformers to adapt to the binding pocket, the present results do not establish a direct relationship between anharmonicity and binding affinity. Rather, the docking results likely arise from a combination of conformational preferences, electronic properties, and specific non-covalent interactions formed within the enzyme active site.

Overall, **structure determines anharmonicity**, which appears to modulate the balance between rigidity and flexibility, both of which may contribute differently to enzyme binding. Increased flexibility may facilitate penetration into the binding pocket and adaptation to different enzyme environments.

Furthermore, hydroxyl-substituted derivatives based on conformations **b** and **c** were designed, and in the case of Protein Kinase C Delta, the docking scores increased by approximately 10%. ADME predictions [43,44], indicated that the proposed molecule is non-toxic and suitable for further experimental and preclinical studies (Tables S1–S3) [13].

Overall, the results suggest that thiocarbohydrazone derivatives represent promising candidates for the development of bioactive compounds. Future work should focus on experimental validation of the predicted spectroscopic data and on in vitro and in vivo studies to confirm the inhibitory activity against Acetylcholinesterase and Protein Kinase C. Moreover, the improved docking scores observed after hydroxyl group substitution indicate that further structural optimization could enhance biological activity. The favorable docking results obtained in this study are consistent with previous experimental reports demonstrating the biological potential of thiocarbohydrazone derivatives, including antiviral activity and potent inhibition of acetylcholinesterase and other metabolic enzymes. These findings support the potential of the investigated molecule as a promising starting point for bioactive compound development. Nevertheless, the docking results should be interpreted as qualitative predictions, and experimental validation is required to confirm the proposed biological activities [10,45].

## Figures and Tables

**Table 1:** Selected harmonic and anharmonic vibrational modes in cm^−1^, corresponding IR intensities (KM/mole), Raman scattering activities (A^4^/amu) and corresponding assignments of conformer **a**.

A/A	Harmonic Vibrational Mode	Anharmonic Vibrational Mode	IR Intensities	Raman Scattering Activities	Vibrational Mode
7	193.7	245.5	17.23	0.54	ρ (NH_2_)
10	283.6	282.3	20.89	9.71	
13	446.4	436.8	7.19	1.00	Wagging of the phenyl ring
15	543.6	503.9	78.36	17.08	δ (N–H)
16	575.1	542.7	99.86	2.97	
17	595.9	589.1	9.42	54.62	
18	618.8	632.5	17.15	3.09	
19	627.9	621.4	2.43	1.59	γ (N–C(S)–N)
20	633.3	624.2	2.38	3.70	Deformation stretches of the phenyl ring
21	699.0	687.7	3.42	5.96	
22	713.3	697.0	45.20	1.42	γ (CHph)
23	768.4	760.7	27.47	9.09	γ (CHph)
24	810.5	792.4	26.32	14.90	
25	862.7	849.4	6.82	9.96	τ (CHph)
26	872.4	838.0	68.30	59.51	
27	897.0	891.3	26.57	35.10	τ (CHph), δ (C–H)
28	951.1	933.5	11.94	0.64	τ (CHph), δ (C–H)
30	995.7	975.9	56.67	5.56	τ (CHph), γ (NH_2_)
32	1014.9	1006.9	0.15	63.47	
33	1049.4	1033.5	8.74	26.02	ρ (CHph)
34	1073.5	1055.8	106.96	46.37	ν (N–NH)
35	1109.8	1081.3	10.12	0.51	δ (CHph)
38	1215.7	1189.1	122.78	62.92	
39	1223.1	1190.4	150.59	48.16	
40	1239.9	1199.7	56.81	17.06	ν_as_ (NH–C(S)–NH–NH_2_)
41	1321.7	1300.1	30.94	24.82	v (C = C) ph
43	1357.9	1343.8	16.61	4.72	ρ (CHph)
44	1377.0	1338.2	123.36	1.05	
45	1405.4	1374.4	13.96	60.13	δ (C–H)
46	1475.6	1447.1	5.30	23.64	
47	1513.8	1474.7	303.20	3.76	
49	1531.7	1476.2	372.08	40.04	ν (C(S)–NH)
51	1637.8	1596.6	3.35	690.49	ν (C = C)ph
52	1658.1	1617.5	16.66	1044.43	ν (CH = N)
53	1706.9	1636.1	36.90	13.78	δ (NH_2_)
54	3139.7	3031.6	6.33	97.97	ν (C–H)
55	3163.3	3011.2	3.14	40.48	ν_as_ (CHph)
57	3179.3	3055.1	9.35	104.34	ν_s_ (CHph), v_as_ (CHph)
58	3187.4	3083.8	14.83	51.11	ν_s_ (CHph), v_as_ (CHph)
59	3195.1	3063.4	9.24	305.78	ν_s_ (CHph)
61	3518.4	3326.9	5.69	42.65	ν_as_ (NH_2_)
62	3534.8	3348.0	25.46	228.68	ν (N–H)
63	3597.6	3417.5	85.68	72.10	ν (N–H)

**Table 2:** Selected harmonic and anharmonic vibrational modes in cm^−1^, corresponding IR intensities (KM/mole), Raman scattering activities (A^4^/amu) and corresponding assignments of conformer **b**.

A/A	Harmonic Vibrational Mode	Anharmonic Vibrational Mode	IR Intensities	Raman Scattering Activities	Vibrational Mode
5	149.2	144.4	5.80	2.05	ρ (NH_2_), τ (NH–NH)
6	177.0	178.5	25.98	5.49	ρ (NH_2_), τ (NH–NH), γ (CH–NH)
7	196.4	249.2	5.78	4.83	ρ (NH_2_), ρ (CHph)
8	209.6	94.5	10.32	4.29	ρ (NH2), γ (CH–NH–NH)
10	300.5	289.5	22.06	4.54	
12	395.4	395.1	14.43	4.54	δ (CH–NH)
14	430.5	356.9	121.82	17.24	ρ (NH_2_), τ (NH–NH)
15	482.5	491.9	138.26	27.38	ρ (NH_2_), γ (NH–NH)
16	520.2	513.5	17.06	0.87	Wagging of the phenyl ring
17	535.2	519.3	21.70	6.25	
20	651.6	643.1	21.62	4.70	
21	700.6	675.9	26.48	15.61	
22	703.8	693.4	40.27	0.04	γ (CHph)
23	752.8	740.5	30.39	8.07	
24	770.9	770.1	36.62	1.98	γ (CHph)
26	894.8	880.4	16.83	13.79	δ (CH–NH)
29	977.8	932.1	97.65	5.59	γ (NH_2_)
32	1014.6	999.8	0.11	120.56	
33	1046.5	1030.6	6.85	18.95	ρ (CHph)
34	1096.9	1071.9	30.08	120.24	δ (CHph)
35	1117.9	1091.0	22.29	174.58	
37	1198.2	1180.8	0.57	188.68	
38	1213.6	1183.3	234.31	320.05	
39	1253.0	1225.5	93.75	109.82	ν_as_ (C–CH = N–NH)
40	1287.2	1246.2	291.85	13.01	ρ (NH–NH)
41	1313.4	1283.2	177.53	19.24	
42	1342.5	1301.8	93.34	90.90	
44	1359.6	1334.2	21.29	32.23	τ (NH_2_)
45	1401.1	1367.1	32.06	16.07	ρ (CH–NH)
46	1478.8	1450.8	9.68	75.41	
47	1495.7	1455.4	177.50	6.34	δ (N–H)
48	1523.7	1489.3	25.68	90.80	
49	1560.9	1518.3	226.90	15.41	δ (N–H)
50	1613.8	1574.0	2.03	324.59	ν_s_ (C = C)ph
51	1639.9	1600.0	0.71	1442.94	
52	1673.0	1635.1	4.79	1906.21	v (C = N)
53	1712.8	1651.5	28.85	16.89	δ (NH_2_)
54	3005.0	2841.0	55.87	71.20	ν (C–H)
55	3155.5	3003.8	7.62	43.35	ν_as_ (CHph)
56	3166.9	3066.3	0.38	89.65	ν_s_ (CHph), v_as_ (CHph)
57	3177.4	3019.3	18.91	109.35	ν_s_ (CHph), v_as_ (CHph)
58	3188.9	3114.6	18.81	260.59	ν_s_ (CHph), v_as_ (CHph)
59	3199.1	3057.1	5.31	102.88	ν_s_ (CHph)
60	3435.9	3217.0	7.29	169.27	ν_s_ (NH_2_)
61	3455.6	3272.8	10.44	372.70	ν (N–H)
62	3518.0	3336.2	11.70	75.89	ν_as_ (NH_2_)
63	3570.7	3405.9	56.80	135.57	ν (N–H)

**Table 3:** Selected harmonic and anharmonic vibrational modes in cm^−1^, corresponding IR intensities (KM/mole), Raman scattering activities (A^4^/amu) and corresponding assignments of conformer **c**.

A/A	Harmonic Vibrational Mode	Anharmonic Vibrational Mode	IR Intensities	Raman Scattering Activities	Vibrational Mode
5	157.3	152.3	6.91	2.47	
7	234.8	212.5	11.92	2.70	
8	259.4	237.5	23.49	1.77	τ (NH_2_)
9	273.8	267.3	20.24	1.89	
10	303.6	325.8	2.24	11.65	
11	327.1	316.5	13.67	1.19	
15	517.8	508.7	26.80	2.64	Wagging of the phenyl ring
16	535.9	525.9	15.23	10.32	
17	626.3	576.2	85.05	109.57	δ (N–H)
19	641.4	616.5	18.80	4.84	
20	686.1	653.3	26.98	41.40	
21	703.0	690.9	41.12	0.67	γ (CHph)
22	721.0	716.9	82.99	2.44	δ (NH–NH)
23	770.4	761.8	35.05	2.05	γ (CHph)
24	820.8	772.3	63.56	13.29	γ (NH_2_)
26	889.3	862.2	58.52	8.57	
28	955.2	923.1	82.43	25.13	
29	981.8	962.4	13.30	3.50	δ (C–H)
32	1014.8	1000.2	0.20	127.92	
33	1045.6	1028.8	7.11	42.67	ρ (CHph)
34	1060.1	1029.8	11.81	213.00	ν (N–NH)
35	1094.1	1062.6	65.40	1.97	
36	1108.3	1080.1	4.21	18.09	δ (CHph)
38	1194.1	1159.9	365,67	109.68	δ (NH–NH)
39	1196.6	1177.6	82.12	79.08	
40	1251.9	1223.5	59.99	341.60	ρ (CHph–CH)
41	1306.1	1264.2	7.53	10.63	τ (NH_2_)
42	1331.3	1301.8	7.18	66.52	ρ (CHph)
44	1391.7	1345.1	301.88	43.41	ν_as_ (N–C(S)–N)
45	1408.6	1374.8	49.47	17.32	ρ (NH–NH–CH)
46	1472.6	1433.4	1.63	176.23	
47	1485.3	1452.6	23.89	4.61	ρ (NH–NH–CH), δ (CHph)
48	1525.4	1491.1	0.91	111.62	
49	1555.4	1506.4	205.46	266.92	δ (NH–NH)
53	1686.5	1654.3	40.01	84.60	δ (NH_2_)
54	3118.5	2961.2	15.44	18.26	ν (C–H)
55	3155.3	3003.9	8.42	29.07	ν_as_ (CHph)
57	3176.2	3082.8	19.74	115.30	ν_as_ (CHph)
58	3188.2	3056.9	20.95	270.47	ν_s_ (CHph), v_as_ (CHph)
59	3200.0	3019.8	5.39	116.06	ν_s_ (CHph)
60	3468.31	3322.9	1.54	211.56	ν_s_ (NH_2_)
61	3525.2	3349.3	36.89	54.44	ν (N–H)
62	3577.6	3396.9	8.71	47.39	ν_as_ (NH_2_)
63	3622.1	3450.9	57.34	190.63	ν (N–H)

**Table 4:** HOMO–LUMO energy difference (ΔE_L-H_ in eV), calculated reactivity descriptors Hardness (η), Softness (σ), Chemical Potential (μ), and Electrophilicity (ω) of the conformers in DMSO solvent and in water solvent.

Conf.	ΔE_L-H_	η	σ	μ	ω	ΔE_L-H_	η	σ	μ	ω
	DMSO					water				
**a**	4.507	2.254	0.444	−4.095	3.720	4.481	2.241	0.446	−4.185	3.908
**b**	4.356	2.178	0.459	−4.037	3.741	4.352	2.176	0.460	−4.171	3.998
**c**	4.172	2.086	0.479	−4.010	3.854	4.106	2.053	0.487	−4.164	4.223

**Table 5:** Overall results of molecular docking with comparison with the known inhibitors.

Compound/Isomer	Target	Docking Score
**1a**	Cathepsin B	−5.00
**1b**	Cathepsin B	−4.77
**1c**	Cathepsin B	−5.21
**2b**	Cathepsin B	−4.82
**2c**	Cathepsin B	−4.77
**Leupeptin**	Cathepsin B	−5.66
**1a**	Acetylcholinesterase	−6.77
**1b**	Acetylcholinesterase	−6.70
**1c**	Acetylcholinesterase	−6.84
**Donepezil**	Acetylcholinesterase	−11.09
**1a**	HER2	−6.61
**1b**	HER2	−7.41
**1c**	HER2	−6.81
**Lapatinib**	HER2	−8.78
**1a**	Protein Kinase C	−5.79
**1b**	Protein Kinase C	−5.88
**1c**	Protein Kinase C	−5.85
**2b**	Protein Kinase C	−6.23
**2c**	Protein Kinase C	−6.30
**Balanol**	Protein Kinase C	−5.62
**1a**	Protein Kinase A	−4.93
**1b**	Protein Kinase A	−5.40
**1c**	Protein Kinase A	−5.04
**Chelerythrin**	Protein Kinase A	−9.39

## Data Availability

The original contributions presented in this study are included in the article and Supplementary Materials. Further inquiries can be directed to the corresponding authors.

## Supplementary Materials

The following supporting information can be downloaded at: https://www.mdpi.com/article/10.3390/ph19071108/s1, Figure S1: NBO Charges in water solventtitle; Table S1: The physicochemical parameters for thiocarbohudrazone; Table S2. The ADME (Absorption, Distribution, Metabolism, Excretion) results of thiocarbohydarzone according to preADMET; Table S3. Toxicity results of the thio-carbohydrazone according to pKCsm; Table S4: Frontier molecular orbital (HOMO and LUMO) energies and calculated global reactivity descriptors of the conformers in the gas phase, in DMSO solvent and in water solvent.
