# Supplementary material for: In Silico Docking and Spectroscopic Evaluation of a Thiocarbohydrazone Derivative: Structural Elucidation and Enzyme Inhibitory Mechanisms
Source: Pharmaceuticals (Basel). 2026 Jul 17;19(7):1108. doi: 10.3390/ph19071108 (PMC13415609; doi:10.3390/ph19071108)

Article

In Silico Docking and Spectroscopic Evaluation of a Thiocarbohydrazone Derivative: Structural Elucidation and Enzyme Inhibitory Mechanisms

Maria Karatzia [1], Nikitas Georgiou [2,*], Ektoras Vasileios Apostolou [3], Eleftherios Papamichalis [4], Sophia C. Hayes [1], Thomas Mavromoustakos [3] and Demeter Tzeli [4,5,*]

1 Department of Chemistry, University of Cyprus, P.O. Box 20537, Nicosia 1678, Cyprus; karatzia.d.maria@ucy.ac.cy (M.K.); charalambous-hayes.sophia@ucy.ac.cy (S.C.H.)
2 Center for Interdisciplinary Biosciences, Technology and Innovation Park, P.J. Safarik University in Kosice, Jesenna 5, 04001 Kosice, Slovakia
3 Laboratory of Organic Chemistry, Department of Chemistry, National and Kapodistrian University of Athens, Panepistimiopolis Zografou, 15771 Athens, Greece; ektorasap@chem.uoa.gr (E.V.A.); tmavrom@chem.uoa.gr (T.M.)
4 Laboratory of Physical Chemistry, Department of Chemistry, National and Kapodistrian University of Athens, Panepistimioupolis Zografou, 11571 Athens, Greece; elpapamich@chem.uoa.gr
5 Theoretical and Physical Chemistry Institute, National Hellenic Research Foundation, 48 Vassileos Constan-tinou Ave., 11635 Athens, Greece
* Correspondence: nikitas.georgiou@upjs.sk (N.G.); tzeli@chem.uoa.gr (D.T.)

Table S1. The physicochemical parameters for thiocarbohudrazone.

Properties	Compound
Molecular Weight	194.26 g/mol
LogP	0.3583
Rotable bonds	2
Hydrogen Bond Acceptors	3
Hydrogen Bond Donors	3
TPSA Surface Area	81.795 (Å^2)
Water solubility	-1.805 (mol/L)

The compound obies in the Lipinski's Rule of Five[55] and Veber's Rule[56], because they have less than 7 rotable bonds. The TPSA values of the compounds were observed in the range of 81.795-117.711 Å2. These values were lower the limit of 160 Å2 underlying a good oral bioavailability. The upper limit for the TPSA for a molecule to penetrate the brain is around 90 Å^2.

Table S2. The ADME (Absorption, Distribution, Metabolism, Excretion) results of thiocarbohydarzone according to preADMET.

	**Compound**
BBB	0.526396
Buffer_solubility_mg_L	292.482
Caco2	14.9063
CYP_2C19_inhibition	Non
CYP_2C9_inhibition	Non
CYP_2D6_inhibition	Non
CYP_2D6_substrate	Non
CYP_3A4_inhibition	Non
CYP_3A4_substrate	Non
HIA	90.293464
MDCK	305.145
Pgp_inhibition	Non
Plasma_Protein_Binding	72.227240
Pure_water_solubility_mg_L	131.444
Skin_Permeability	-2.86899

According to preADMET, the BBB[57] value is less than one. As a result, **it** is classified as inactive in Central Nervous System (CNS). The value for Human intestinal absorption is high, and this signifies that this compound might be better absorbed from the intestinal tract on oral administration. It is not inhibitor of CP isoenzymes and therefore are not toxic

Table S3. Toxicity results of the thiocarbohydrazone according to pKCsm.

**Properties**	**Compound**
Toxicity	
AMES toxicity	Yes
Max. tolerated dose (human)	1.27 (log mg/kg/day)
Herg I inhibitor	No
Herg II inhibitor	No
Oral Rat Acute Toxicity (LD50)	2.928 (mol/kg)
Oral Rat Chronic Toxicity	1.839 (log mg/kg_bw/day)
Hepatotoxicity	No
Skin Sensitisation	Yes

According to pkCSm, it has not been predicted to be hepatotoxic. It has negative AMES[58] toxicity and as a result it is not mutagenic.

Table S4. Frontier molecular orbital (HOMO and LUMO) energies and calculated global reactivity descriptors of the conformers in the gas phase, in DMSO solvent and in water solvent.

Conf.		HOMO (eV)	LUMO (eV)	ΔE(L-H)	Hardness (η)	Softness (σ)	Chemical Potential (μ)	Electrophilicity (ω)
**a**	In gas phase	-6.086	-2.027	4.059	2.030	0.493	-4.057	4.054
**b**	In gas phase	-5.989	-1.925	4.064	2.032	0.492	-3.957	3.853
**c**	In gas phase	-5.932	-1.999	3.933	1.967	0.509	-3.966	3.998
**a**	In DMSO	-6.348	-1.841	4.507	2.254	0.444	-4.095	3.720
**b**	In DMSO	-6.215	-1.859	4.356	2.178	0.459	-4.037	3.741
**c**	In DMSO	-6.096	-1.924	4.172	2.086	0.479	-4.010	3.854
**a**	In WATER	-6.425	-1.944	4.481	2.241	0.446	-4.185	3.908
**b**	InWATER	-6.348	-1.995	4.352	2.176	0.460	-4.171	3.998
**c**	In WATER	-6.218	-2.111	4.106	2.053	0.487	-4.164	4.223

Figure S1: NBO Charges in water solvent

Conformer a

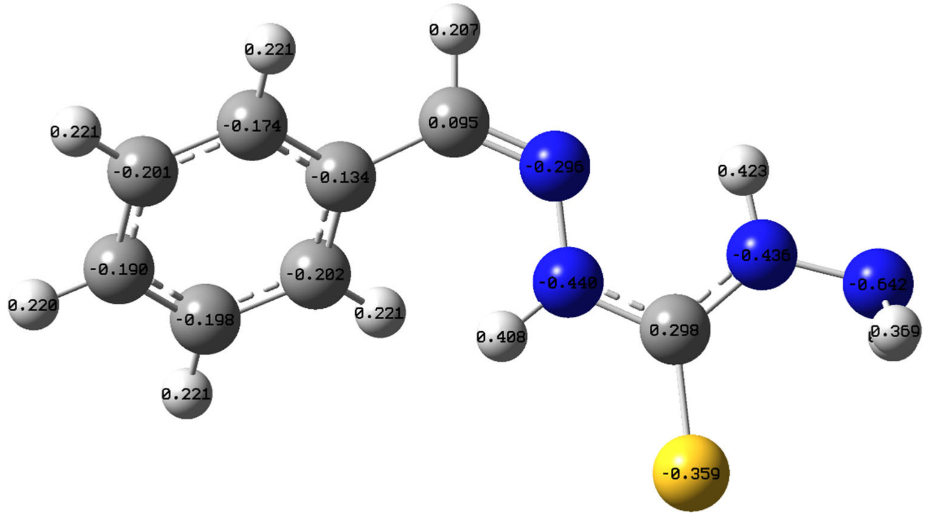

Conformer b

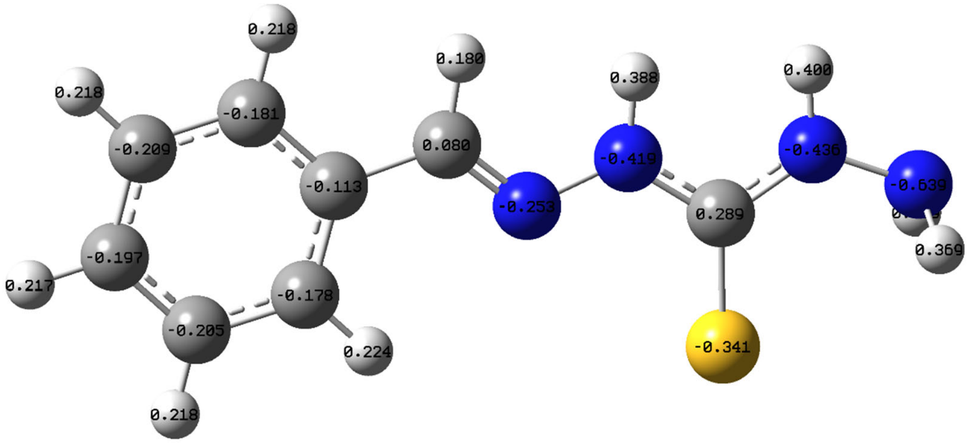

Conformer c

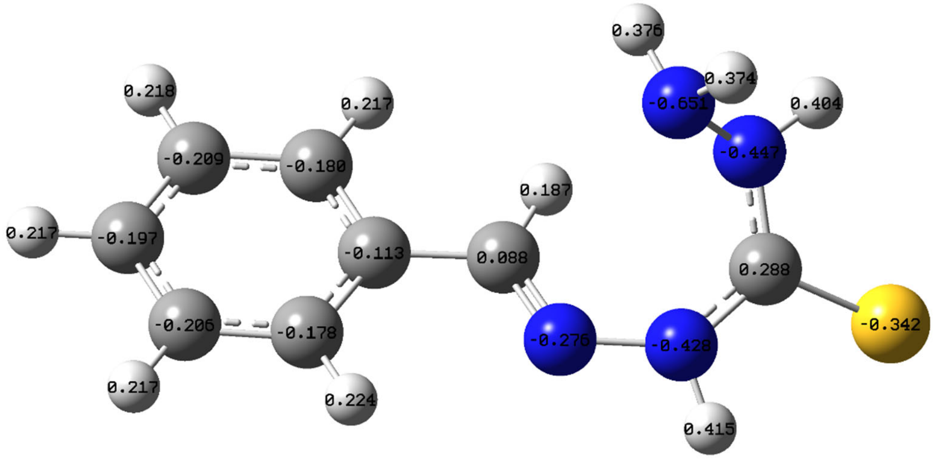

Supplement: Supplementary file 1 [file pharmaceuticals-19-01108-s001.zip › pharmaceuticals-4393726-supplementary.pdf]
